# Microstructure and Enhanced Properties of Copper-Vanadium Nanocomposites Obtained by Powder Metallurgy

**DOI:** 10.3390/ma12030339

**Published:** 2019-01-22

**Authors:** Yong Wang, Jinguo Wang, Haohao Zou, Yutong Wang, Xu Ran

**Affiliations:** 1 Key Laboratory of Automobile Materials, Ministry of Education, Department of Materials Science and Engineering, Jilin University, No. 5988 Renmin Street, Changchun 130025, China; gxgy9406@163.com (Y.W.); mason1006@163.com (J.W.); 2 Key Laboratory of Advanced Structural Materials, Ministry of Education, Changchun University of Technology, No. 2055 Yanan Street, Changchun 130012, China; haoozou@163.com (H.Z.); wyt9408@163.com (Y.W.)

**Keywords:** copper, nanocomposite, high strength, conductivity, strengthening mechanism

## Abstract

Cu-2.4 wt.%V nanocomposite has been prepared by mechanical alloy and vacuum hot-pressed sintering technology. The composites were sintered at 800 °C, 850 °C, 900 °C, and 950 °C respectively. The microstructure and properties of composites were investigated. The results show that the Cu-2.4 wt.%V composite presents high strength and high electrical conductivity. The composite sintered at 900 °C has a microhardness of 205 HV, a yield strength of 404.41 MPa, and an electrical conductivity of 79.5% International Annealed Copper Standard (IACS); the microhardness and yield strength reduce gradually with the increasing consolidation temperature, which is mainly due to the growth of copper grain size. After sintering, copper grain size and V nanoparticle both maintain in nanoscale; the strengthening mechanism is related to grain boundary strengthening and dispersion strengthening, while the grain boundary strengthening mechanism plays the most important role. This study indicates that the addition of small amounts of V element could enhance the copper matrix markedly with the little sacrifice of electrical conductivity.

## 1. Introduction

In recent years, with the rapid development of the aerospace industry, micro-electronics, communication, medicine, life sciences, and so on, there is a high request for copper alloy’s various indicators and the capacity of adapting to an environment, such as possessing high strength and high conductivity simultaneously [1,2,3,4]. It is hard to improve the strength of copper, which always comes at the price of conductivity reduction. How to enhance the strength sharply while keeping the high conductivity of copper as far as possible becomes the important issue in the development of the modern copper processing industry [5]. One of the most common methods to improve the mechanical properties of copper is to add solid solution elements, such as Ti [6], Ni [7], Si [8], and Cr [9] etc. Although this sort of alloy elements could endow copper alloys with higher strength, their conductivity is typically reduced. The strength of some could be more than 1000 MPa, but their conductivity is less than 20% IACS. Therefore, new copper alloys with superior mechanical properties and high conductivity still need to be researched.

It is common knowledge that the alloy elements dissolving in or reacting with the matrix could reduce the conductivity of the matrix. So the element that is mutually insoluble or slightly soluble with copper in the solid state has a uniquely high combination of mechanical properties, electric conductivity, and thermal conductivity, such as Fe [10], Ag [11,12], and Nb [13] etc. Among them, the Cu–Nb system has the best combination of properties. Lei et al [14] prepared nanocrystalline Cu-10 wt.% Nb alloy with high strength and high conductivity by mechanical alloy method (MA) and vacuum hot-pressed sintering technology. The tensile strength of as-received sample is 1102 MPa while the conductivity is 57% IACS. Compared with Nb, V possesses higher hardness, low density, and low activation while it also has no intermediate intermetallic phases and a minimum value of mutual solubility in copper [15]. Therefore, V was chosen to enhance copper matrix in this study. Unfortunately, few studies on Cu–V alloy have been reported and most of these researches mainly focus on the Cu–V alloy wire by cold drawing and Cu–V multilayer films [16,17,18,19]. The vanadium fibre reinforced copper matrix wire presents very high strength, but like the Nb and Ag fibre, the V fibre would be divided and spheroidality, which could cause a sharply drop in strength. Therefore, researching the properties of Cu–V nanocomposites is very necessary and the work on this aspect hasn’t been reported yet.

In this study, Cu-2.4 wt.%V nanocomposites were prepared by MA and vacuum hot-pressed sintering technology. The main purpose of this job is to evaluate the effect of consolidation temperature on the microstructure and properties and discuss the strengthening mechanism of composites systematically.

## 2. Materials and Methods

The Cu–V nanocomposites with 2.4 wt.% V were fabricated by MA and vacuum hot-pressed sintering technology. The raw materials used in this study were ultra-fine copper powder (4–7 μm, the purity ≥ 99.9%) and vanadium powder (10–15 μm, the purity ≥ 99.6%). The MA process was carried in a dual-tank three dimensional oscillating high energy ball mill (home-made) with Cr_12_MoV containers and GCr_15_ bearing steel balls under an argon atmosphere. The ball-to-powder weight ratio was 10:1; rotation speed was 370 r/min; the duration of the ball mill was 60 h and acetone was used as process control agent. The milled Cu–V powder was annealed in a hydrogen atmosphere at 560 °C for 1 h to reduce the inner strain and oxygen in Cu–V powder. Then the annealed powder was sintered by vacuum hot pressing sintering under a load of 50 MPa for 2 h at 800 °C, 850 °C, 900 °C, and 950 °C respectively. 

The density was measured by the Archimedes method; X-ray diffraction (XRD) profiles of the samples were analysed using a DMAX2000 X-ray diffractometer (Rigaku Corporation, Beijing, China) with CuKα radiation and the scans of 2θ were taken from 40° to 80°; the microstructure was observed by the EIPHOTO NIKON 300 optical microscope (OM) (Nikon, Shanghai, China), JMS-5500LV scanning electron microscope (SEM) (JMS Corporation, Shenzhen, China), and high resolution transmission electron microscopy (HRTEM) (FEI Corporation, Shanghai, China); TEM samples were prepared using 980 Microconcave grinder and Gatan691 ion milling (GATAN, Shanghai, China). The micro-hardness of the samples was measured on a FM-700 microhardness tester (FT Corporation, Xi’an, China) using a load of 25 gf and a holding of 15 s. The electrical conductivity of the samples was measured on a sigma 2008 digital eddy metal conductance meter (Xiamen tianyan instrument co., LTD., Xiamen, China) at the room temperature. The strength of the samples was tested on a WDW-200 tensile testing machine (Shanghai precision instrument co., LTD., Shanghai, China) using a strain rate of 0.2 mm/min. The tested samples were in the form of a cylindrical pin with 3 mm of diameter and 6 mm of length.

## 3. Results

### 3.1. Phase and Morphology of Samples

**Figure 1:**
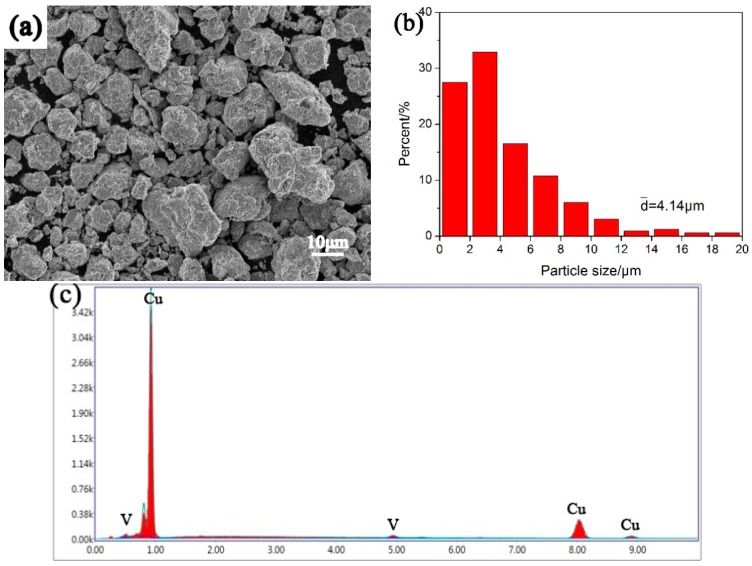
Scanning electron microscope (SEM) image (**a**) and the size distribution; (**b**) of the Cu–V composite power; (**c**) the energy dispersive spectrometer (EDS) result (point scanning) from certain particles; the “k” on y-axis means thousand.

The image of Cu–V composite powder after ball milling for 60 h is shown in Figure 1a. The powder particles show subsphaeroidal and some big particles are found which is due to the welding-on. The particle size distribution is shown in Figure 1b. Mainly, the size of the particle is about several microns and its average size is 4.14 μm. Figure 1c shows the energy dispersive spectrometer (EDS) result from certain particles. It can be observed that V peaks are very weak, which is because vanadium addition is small. EDS (point scanning) was carried out on several particles selected randomly. Three results of them are displayed in Table 1. The mass fractions of vanadium in copper are 2.51%, 2.12%, and 2.24% respectively and very close to 2.4% (the raw material ratio), which indicates that the distribution of vanadium in copper is uniform after 60 h ball milling.

**Figure 2:**
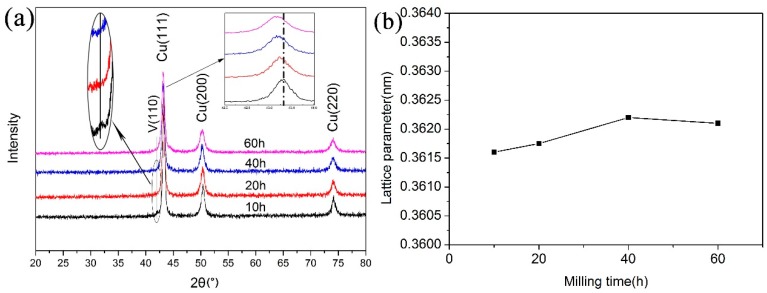
X-ray diffraction (XRD) patterns of Cu–V composite powder (**a**) and the lattice parameter of copper at different milling time (**b**).

To research the existential state of vanadium in copper, XRD analysis was performed on the composite powders after different milling times. The result is shown in Figure 2a. It can be distinctly seen that the peak of copper become weak and wide with the ball milling process, which is due to the grain refining of copper. Furthermore, it can also be found that the Cu peaks move towards low angles with ball milling process (the insert in Figure 2a). Because vanadium addition is small, the peak of vanadium is very weak and only can be detected in composite powders milled for below 10h and then disappears gradually with the ball milling process. The lattice parameters of copper at different milling times are recorded and shown in Figure 2b. In a certain duration of ball milling, the lattice parameter of copper increases with the ball milling process (0–40 h) and then decreases somewhat, which indicates that vanadium dissolves into copper during the ball milling process. The atomic radius of vanadium (0.132 nm) is slightly larger than copper (0.128 nm). When vanadium atoms dissolve into the copper lattice, the copper lattice will expand, which results in the increase of copper lattice parameter and the lattice distortion of copper. As for the later decrease of the copper lattice parameter, it is widely believed today that the reason for this phenomenon is that supersaturated vanadium in copper precipitates out due to the temperature rise after a long time ball milling [20]. From the XRD results, there is no obvious CuO peak, which also indicates that the oxygen contamination is little during the ball milling process.

**Figure 3:**
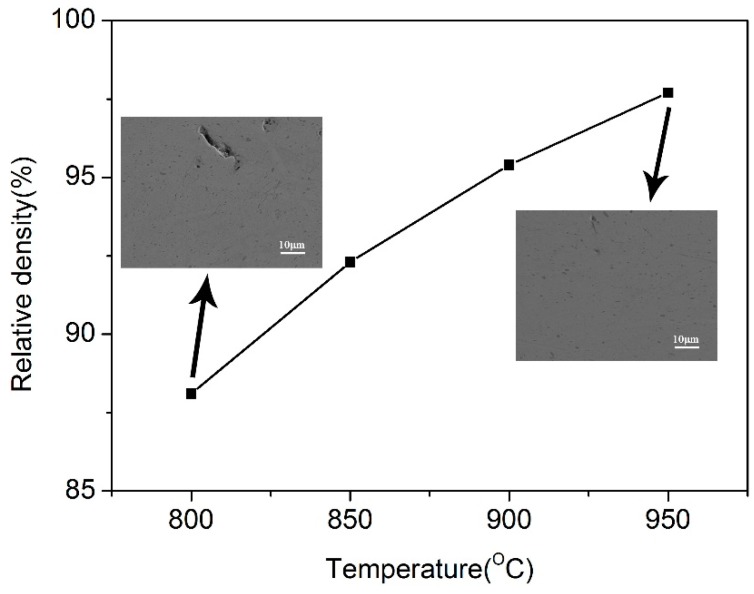
The change of relative density of composites with the consolidation temperatures. In the inset, SEM images show the microstructure of the composite sintered at 800 °C (left) and 950 °C (right).

After ball milling, the composite powders were sintered by vacuum hot pressing at a load of 50 MPa for 2 h at different temperature. The variation of relative density and the corresponding SEM images of the composites versus sintering temperature is shown in Figure 3. It can be seen that the relative density of composite sintered at 800 °C is about 88%, whereas an increase of the consolidation temperature to 950 °C lead to a density of 97%. In the case of the sample sintered at 800 °C, numerous pores can be found. When the sintering temperature increases to 950 °C, few pores can be detected, which is related to the higher density.

**Figure 4:**
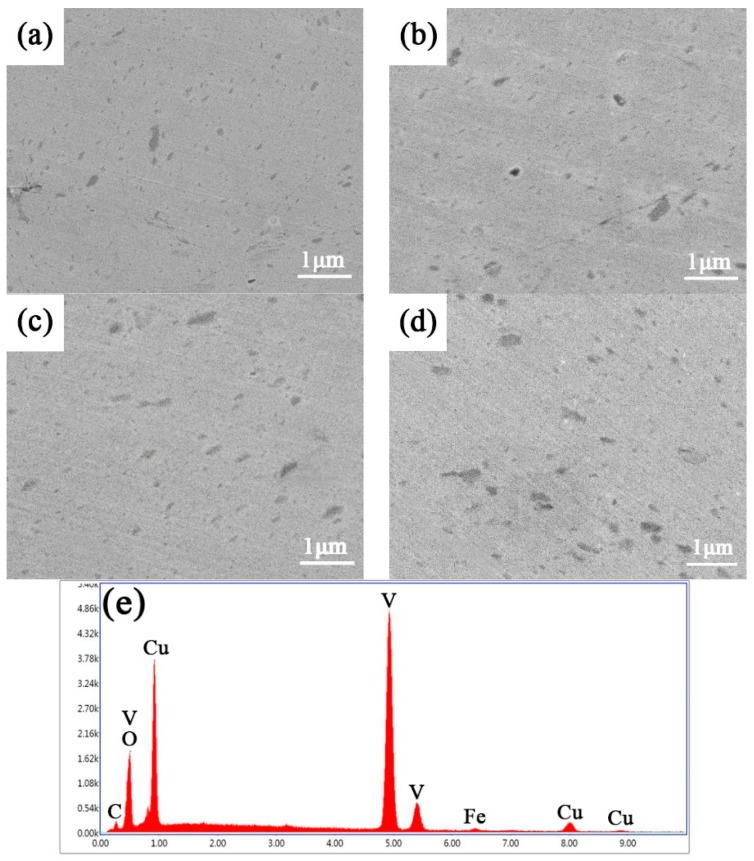
SEM images of Cu/V composites sintered at different temperature: (**a**) 800 °C; (**b**) 850 °C; (**c**) 900 °C; (**d**) 950 °C; (**e**) EDS result of selected V particle in (**d**); the “k” on y-axis means thousand.

Figure 4 shows the SEM images of composites sintered at different temperature. The dark grey particles in the matrix are V nanoparticles, which can be testified by EDS shown in Figure 4e. V nanoparticles are distributed in the copper matrix with a very wide size range after sintering. Nevertheless, there is still an amount of V nanoparticles which are too small to be detected by SEM (the TEM image can verify it shown in later), and only the larger part of V particles can be observed. With the increase of sintered temperature, the quantity of coarse V particles goes up. Therefore, the higher sintering temperature can result in the coarsening of V particles and copper crystal grain, but lower temperature is not good for the densification of composites. From the EDS result, it can be found that a small quantity of Fe is detected, which is introduced during the ball milling process.

**Figure 5:**
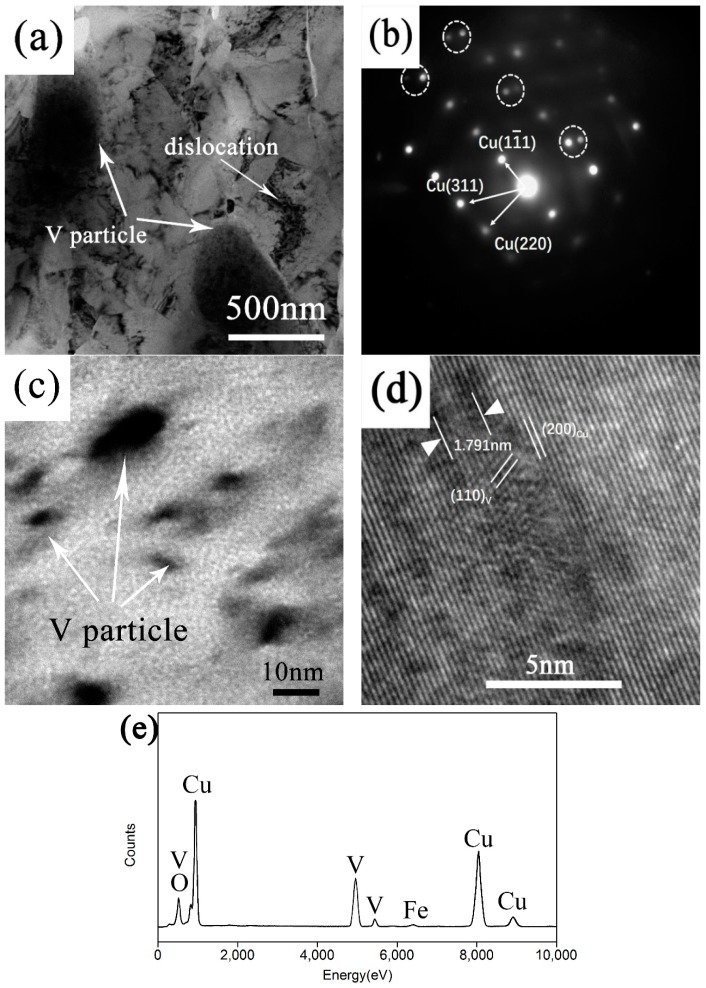
(**a**) TEM image of composite sintered at 900 °C; (**b**) SAED pattern of copper matrix in (**a**); (**c**) BF image showing the nanosized V particles below 10 nm; (**d**) HRTEM image of the interface between V and Cu and (**e**) the EDS result of the V particle in (**c**).

Figure 5a shows the TEM image of microstructure of composite sintered at 900 °C for 2 h. It can be seen that the copper matrix is showing a coarse structure and the average copper crystal grain is larger than 100 nm. Several big V particles are detected which are about several hundred nanometers in size and the interface of V and Cu is tight and no gap is found. The amount of dislocation is also found in the copper matrix (marked by the white arrow). Figure 5b shows the SAED pattern of the copper matrix. It can be seen around the diffraction spots of the copper matrix, there are distributed extra diffraction spots with slightly darker contrast (marked by white circles). According to the similar study [21], the emergence of extra diffraction spots is due to the precipitation of V element from copper matrix. Under higher magnification, the smaller V nanoparticles (testified by EDS shown in Figure 5e) are detected with a size below 10 nm dispersed within the copper matrix, which is shown in Figure 5c. Figure 5d shows the HRTEM image of the interface between V and Cu under the [$\overset{-}{1}$12] zone axis of the copper matrix. It can be seen that V precipitates show parallel moire fringe in HRTEM. The measured fringe interval is 1.791 nm and its direction is parallel to the (200) plane of the matrix. The moire fringe is generated by interference between diffraction beams of two crystals with different lattice constants. Moire fringe interval (L) is the reciprocal of $\vec{\Delta a}$ which is the difference of vector diffraction $\vec{a_{1}}$ and $\vec{a_{2}}$, the direction of fringe is perpendicular to $\vec{\Delta a}$ and can be expressed as [22]

(1) $$L=\frac{1}{|\vec{\Delta a}|}=\frac{d_{1}d_{2}}{\sqrt{d_{1}^{2}+d_{2}^{2}-2d_{1}d_{2}\cos\theta }}$$

where $\vec{a_{1}}$ is the direction of (200)_Cu_, $\vec{a_{2}}$ is the direction of (110)_V_, $d_{1}$ is the interplanar distance of $\vec{a_{1}}$, $d_{2}$ is the interplanar distance of $\vec{a_{2}}$, and θ is the angle between $\vec{a_{1}}$ and $\vec{a_{2}}$. $d_{1}$, $d_{2}$, and θ all can be measured and their value is 2.412 nm, 2.093 nm, and 67.6° respectively. Putting them into formula (1), the Moire fringe interval can be calculated and the result is 1.73 nm which is close to the measured value (1.791 nm). Observed under the HRTEM, the interface between V nanoparticle and matrix is clean and combined well.

It should be noted that the size of V nanoparticles presents a bimodal distribution after sintering according to the SEM and TEM observations. The range of V particle size is from several nanometers to hundreds of nanometers. According to Benghalem and Morris’s view [23], the growth of V particles with a size smaller than 50 nm is due to the volume diffusion coarsening and the larger V particle is controlled by grain boundary diffusion.

### 3.2. Properties of Samples

**Figure 6:**
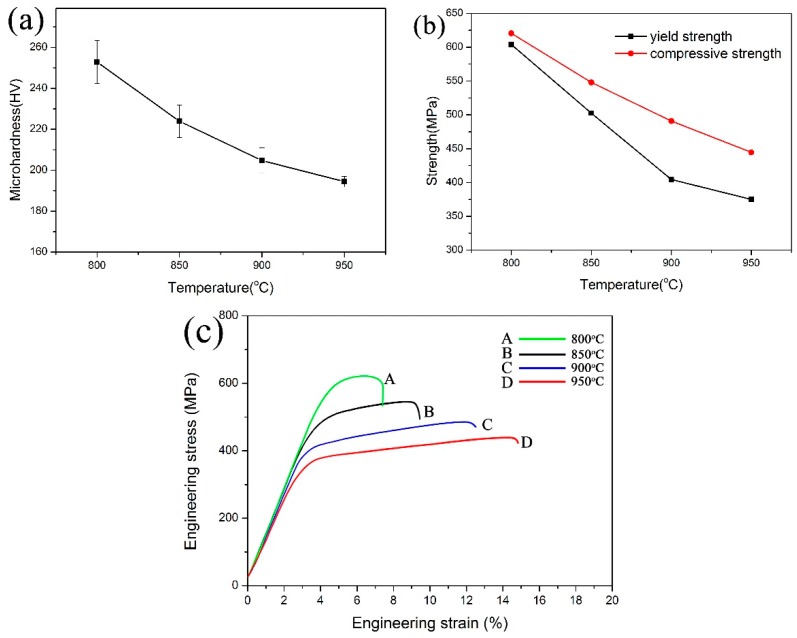
The changes of microhardness (**a**), yield strength and compressive strength (**b**) of composites with the sintering temperature; (**c**) engineering stress–strain curves of composites.

**Figure 7:**
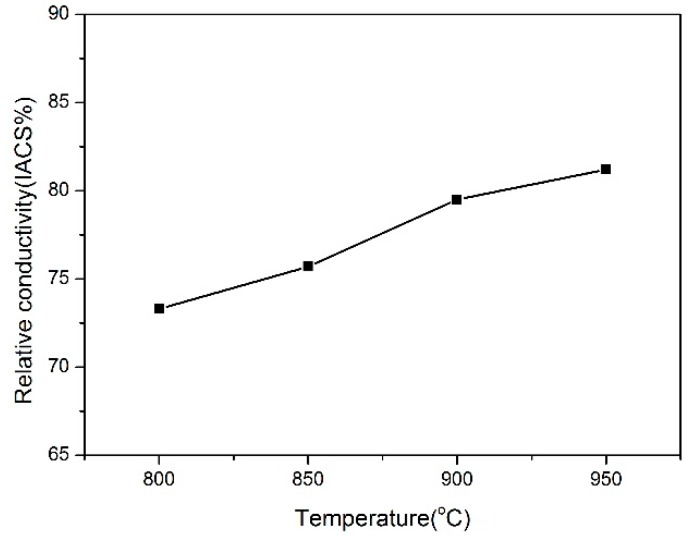
Conductivities of Cu–V composites sintered at different temperatures.

Figure 6a,b shows the variation of microhardness, yield strength, and compressive strength of samples with the sintering temperature. It can be seen that increasing the sintering temperature could lead to the reduction of microhardness and the strength level. However, the sample sintered at 950 °C for 2 h still presents a high microhardness of 194 HV and a high yield strength of 375 MPa. The engineering stress–strain curves of composites are shown in Figure 6c. It can be found that the toughness of composites increases with the sintering temperature, which is due to the increasing relative density. Figure 7 shows the conductivity of Cu–V composites sintered at different temperatures. It can be found that the conductivity of Cu–V composite increases with the increasing consolidation temperature, which is related to the increase of density of composite, growth of copper grain during the sintering, and the precipitation of V atoms from the copper matrix. These factors all can reduce the scattering of electrons and enhance the electrical conductivity. The conductivity of Cu–V composite increases from 58.3% to 81.2% after increasing the temperature to 950 °C. Therefore, Cu-2.4 wt.% possesses high strength and high conductivity simultaneously.

## 4. Discussion

Following is the discussion of the strengthening mechanism of the Cu–V composite. It is known that the property of metallic materials is mainly controlled by its microstructure, such as grain size, dislocations, second phases, solute atoms, and density, etc. for the Cu–V composite in this study. Because V has no intermediate intermetallic phases and a minimum value of mutual solubility in copper matrix and can separate out easily at a relative high temperature, which could be verified by the clean lattice fringe of copper matrix (shown in Figure 5d), so the solution strengthening can also be neglected. The internal strain is decreased to a minimum level after sintering. Therefore, the strain strengthening is not significant here as well. However, the amount of dislocations is found existing in the Cu–V composite (shown in Figure 5a). The dislocations effect each other and plenty of dislocations tangle and form a dislocation wall. V nanoparticles could hinder the movement of dislocation, so the precipitation strengthening via the Orowan mechanism should be considered. Because the V particle possesses very high hardness, it is difficult to shear by dislocations. So the Orowan mechanism of a dislocation–particle interaction mechanism is reasonable. The strength of $\Delta \sigma_{Orowan}$ can be expressed as [24]

(2) $$\Delta \sigma_{Orowan}=\frac{0.81\mathrm{MGb}}{2\pi {\left(1-\nu \right)}^{1/2}}\frac{\ln\pi r/4b}{r\left({\left(3\pi /2f_{V}\right)}^{1/2}-\pi /4\right)}$$

where G is the shear modulus of copper (45.5 GPa), M is the Taylor factor of copper (3.1), b is the Burgers vector of dislocations in copper (0.255 nm), ν is the Poisson’s ratio of copper (0.34), r is the V particle radius, $f_{V}$ is the volume fraction of the V particle. According to the previous research [23], the strengthening effect of the particle with the size above 50 nm is little and can be neglected. However, the $f_{V}$ of V particles with size below 50 nm are difficult to count in TEM images because of the overlap and uncertainty of the thickness of the observation area. Allowing for the average size and the flat area of V particles with size above 50 nm can be measured from the SEM images and their volume fraction can be calculated subsequently. The total volume fraction of V particles is known. So the $f_{V}$ of V particles with size below 50 nm can be figured out. Counting and calculating, the $f_{V}$ of V particles with size below 50 nm in composite sintered at 800 °C, 850 °C, 900 °C, and 950 °C is 2.1%, 1.9%, 1.4%, and 1.2% respectively. The average size of V nanoparticle in the composite sintered at different temperature counted from TEM images is shown in Table 2. Taking these data into the formula (2), the value of $\Delta \sigma_{Orowan}$ can be calculated. The result is displayed in Table 2.

Comparing with the yield strength measured by experiment, the contribution of $\Delta \sigma_{Orowan}$ is not the main. So there must be other strengthening mechanism. Considering the grain boundary strengthening, the copper grain size was measured by XRD. The average copper grain size (shown in Table 2) for composite sintered at different temperature is 138 nm, 186 nm, 264 nm, and 285 nm respectively. It can be seen that higher sintering temperature could lead to the coarsening of copper grain size dramatically, which is because the low content of V nanoparticles has limited hindering effects on copper grain growth. Generally, the contribution of fine crystal reinforcing should be analyzed based on the Hall–Petch relation [25]. Namely,

(3) $$\sigma_{H-P}=\sigma_{0}+kd^{-1/2}$$

where $\sigma_{0}$ is strength constant which controlled by grain structure and dislocation density, k is Hall–Petch coefficient, and d is grain size. Based on the Lei et al’s report [14], the k of nano-crystalline copper is 4.0 × 10^3^ MPa/nm^1/2^ and the $\sigma_{0}$ is 29 MPa. Through calculating, the results are shown in Table 2.

Through the above discussion, it can be found that the strength of Cu–V composite is related to the grain boundary strengthening produced by nano-crystalline grains and the dispersion strengthening produced by V nanoparticles. It should be noted that the grain boundary strengthening mechanism plays the most important role.

## 5. Conclusions

In this study, Cu-2.4 wt.%V nanocomposite has been prepared by mechanical alloy and vacuum hot-pressed sintering technology. The microstructure and properties have been observed and analysed. The conclusions are as follows.

(1) With the increasing sintering temperature, the density and conductivity of Cu-2.4 wt.%V composite increase, while the microhardness and yield strength reduce gradually, which is mainly due to the growth of copper grain size.

(2) Cu-2.4 wt.%V nanocomposite presents high strength and high electrical conductivity. The composite sintered at 900 °C has a microhardness of 205 HV, a yield strength of 404.41 MPa, and an electrical conductivity of 79.5% IACS.

(3) After sintering, copper grain size and V nanoparticle both maintain in nanoscale; the strengthening mechanism is related to the grain boundary strengthening and dispersion strengthening, while the grain boundary strengthening mechanism plays the most important role.

## Figures and Tables

**Table 1:** V content of selected particles.

Sample	Mass (%)	Atom (%)
1	2.51	3.11
2	2.12	2.62
3	2.24	2.76

**Table 2:** Results of calculative $\Delta \sigma_{Orowan}$ and $\sigma_{H-P}$.

Consolidation Temperature (°C)	Copper Grain Size (nm)	V Nanoparticle Size (nm)	V Nanoparticle Content (vol.%)	$\Delta \sigma_{Orowan}\;(\mathrm{MPa})$	$\sigma_{H-P}\;(\mathrm{MPa})$	Yield Strength (MPa)
800	138	8.5	2.1	243.36	369.50	603.96
850	186	10.4	1.6	185.77	322.29	502.55
900	264	12.1	1.2	144.85	275.18	404.41
950	285	13.8	0.9	114.63	265.94	374.84
